# Factors associated with thrombotic disease in dogs with renal proteinuria: A retrospective of 150 cases

**DOI:** 10.1111/jvim.16973

**Published:** 2023-12-26

**Authors:** Luca Fortuna, Harriet M. Syme

**Affiliations:** ^1^ Department of Clinical Science and Services The Royal Veterinary College Hatfield United Kingdom

**Keywords:** cardiology, cardiovascular, kidney, protein losing nephropathy, pulmonary thromboembolism, renal/urinary tract, respiratory tract, thromboembolism

## Abstract

**Background:**

Knowledge of additional risk factors for thrombotic disease (TD) among dogs with renal proteinuria is limited; these might differ for TD affecting the systemic arterial (AT), systemic venous (VT), and pulmonary circulation (PT).

**Hypothesis/Objectives:**

To compare signalment and clinicopathological data between dogs with renal proteinuria with or without TD, and between dogs with AT, VT, and PT.

**Animals:**

One hundred fifty client‐owned dogs with renal proteinuria, 50 of which had TD.

**Methods:**

Retrospective case‐controlled study. A database search (2004‐2021) identified proteinuric dogs (UPC > 2) with and without TD. Clinicopathological data were obtained from the records. TD and non‐TD (NTD) groups were compared by binary logistic regression, and AT, VT, and PT groups by multinomial regression. Normal data presented as mean ± SD, non‐normal data presented as median [25th, 75th percentiles].

**Results:**

Cavalier King Charles Spaniels were overrepresented in the TD group (OR = 98.8, 95% CI 2.09‐4671, *P* = .02). Compared to NTD cases, TD cases had higher concentration of neutrophils (11.06 [8.92, 16.58] × 10^9^/L vs 7.31 [5.63, 11.06] × 10^9^/L, *P* = .02), and lower concentration of eosinophils (0 [0, 0.21] × 10^9^/L vs 0.17 [0.04, 0.41] × 10^9^/L, *P* = .002) in blood, and lower serum albumin (2.45 ± 0.73 g/dL vs 2.83 ± 0.73 g/dL, *P* = .04). AT cases had higher serum albumin concentrations than VT cases (2.73 ± 0.48 g/dL vs 2.17 ± 0.49 g/dL, *P* = .03) and were older than PT cases (10.6 ± 2.6 years vs 7.0 ± 4.3 years, *P* = .008). VT cases were older (9.1 ± 4.2 years vs 7.0 ± 4.3 years, *P* = .008) and had higher serum cholesterol concentration (398 [309‐692 mg/dL] vs 255 [155‐402 mg/dL], *P* = .03) than PT cases.

**Conclusions and Clinical Importance:**

Differences between thrombus locations could reflect differences in pathogenesis.

**Abbreviations**
AT: arterial thrombotic disease
NTD: no thrombotic disease
PLN: protein losing nephropathy
PT: pulmonary thrombotic disease
TD: thrombotic disease
UPC: urine protein to creatinine ratio
VT: venous thrombotic disease

## 1 INTRODUCTION

Protein losing nephropathy (PLN) occurs when damage to the glomerular filtration barrier leads to plasma proteins crossing into the filtrate in excessive quantities and being lost in the urine.^1^ Dogs with PLN are hypercoagulable in vitro,^2^ and at risk of thrombotic disease (TD),^3^, ^4^ but there is limited work exploring additional risk factors for TD among dogs with PLN. It could be important to identify dogs at high risk so that they can receive targeted anti‐thrombotic therapy. In veterinary medicine, there is currently little direct evidence for the benefit of anti‐thrombotics in dogs.^5^

Two prospective studies in dogs with PLN have explored associations between clinicopathological parameters and in‐vitro assessment of hypercoagulability using thromboelastography.^2^, ^6^ Although correlations were not noted overall, the dogs with documented TD in 1 of the studies had lower serum albumin concentrations and higher urine protein: creatinine (UPC) ratios than did unaffected dogs.^2^ Comparisons were limited by small numbers of cases developing TD in either study. Thromboelastography is not widely available in clinical practice, which limits the application of these findings. Dogs with PLN have higher protein C levels compared to normal dogs, which is of uncertain clinical importance.^7^

PLN in dogs is most commonly described to cause TD in the pulmonary vasculature (PT).^4^ In humans, pulmonary thrombi are considered to be venous in origin,^8^ but in small animals a distinction between primary pulmonary thrombi and thromboemboli from the systemic venous circulation is rarely made, and primary venous thrombi might be less common than in humans.^9^ AT are reported in dogs with PLN.^10^, ^11^ If different risk factors were identified for developing PT, VT, and AT within the population of dogs with PLN it could in future help target optimal prophylactic treatment. There are theoretical reasons to select anticoagulants in diseases at risk of VT and anti‐platelet drugs in diseases at risk of AT, although in humans “crossover” protection has been demonstrated in conditions associated with thrombosis and so optimum thromboprophyaxis likely depends on more than simply the sites at risk of being affected by TD.^12^

The objectives of this retrospective study were to determine if clinicopathological or signalment variables differ between dogs with proteinuria of renal origin with or without TD, and if any clinicopathological or signalment variables differ between dogs with proteinuria of renal origin and thrombi in different locations (PT, VT, or AT).

## 2 METHODS

A retrospective case‐controlled study was designed. Dogs were included in the TD group if they had UPC > 2 in the absence of pyuria (>5 leukocytes per high powered field), bacteriuria, pigmenturia or gross hemorrhage. Dogs with proteinuria and TD that had presented to the Queen Mother Hospital for Animals between January 1, 2004 and January 1, 2021 were identified through a search on the Vetcompass database for appropriate combinations of the terms “Protein losing nephropathy,” “PLN,” “glomerulopathy,” “glomerulonephritis,” “amyloidosis,” “proteinuria,” “proteinuric,” “thrombus,” “thrombosis,” and “thromboembolism.” Cases were included if they met the following criteria:

- Urine protein to creatinine ratio (UPC) > 2.
- Thrombus visualized by diagnostic imaging (ultrasound/CT/MRI), during post mortem examination, or where suggestive (but not conclusive) imaging findings were present and alternative diagnoses were excluded; for example, acute onset dyspnea with unremarkable thoracic radiographs, pulmonary hypertension demonstrated on echocardiography or hypoxemia.

These cases were further classified by thrombus location as having either VT, AT, or PT. Cases with thrombi in more than 1 location were excluded from the comparison by thrombus location but included in the comparison with NTD cases.

Dogs with proteinuria but without TD were selected for comparison by locating the TD cases on a chronological list of all UPC results run by the laboratory and selecting the preceding and subsequent case that met the urinalysis criteria for renal proteinuria (ie, UPC > 2 and absence of infection or gross hemorrhage) but for which there was no clinical suspicion of TD.

In all cases where more than 1 visit had been made to the hospital, data were taken from the earliest visit where the above urinalysis criteria were met. Signalment variables (age, breed, sex, neuter status) and comorbidities were recorded, as were UPC, noninvasive blood pressure (NIBP), and selected routine hematology and biochemistry results where available. Platelet counts were not included in the analysis because in the majority of cases this was considered an under‐estimate because of clumping and so the numerical count was therefore unreliable, but the presence or absence of platelet clumps on blood smear examination were recorded. Breeds that were represented by fewer than 5 cases were grouped into “Small other breed” and “Large other breed” based on whether the expected adult weight of the breed was less than or greater than 15 kg respectively, and sighthound breeds were combined into 1 group.

All statistical analysis were performed using commercially available software (IBM SPSS Statistics for Windows, Version 27.0. Armonk, New York, IBM Corp). Breed, sex, neuter status, presence of platelet clumps on blood smear, history of ongoing oral administration of corticosteroids and any comorbidity present in ≥5 dogs in the TD group (with hyperadrenocorticism also specifically included because of a previous association with both TD and proteinuria)^4^ were compared between TD and NTD cases, and between cases with VT, AT, and PT using the Fisher's exact test. Age, UPC, NIBP, and results of routine biochemistry and hematology were assessed for normality using the Shapiro Wilk test. Normally distributed data are presented as mean ± SD, non‐normally distributed data presented as median [25th, 75th percentiles]. Normally distributed variables were compared between groups using the independent T test for the TD versus NTD comparisons, and a 1‐way ANOVA with a Tukey post‐hoc comparison for thrombus location. Non‐normally distributed variables were compared between groups using the Mann‐Whitney *U* test for the TD versus NTD comparisons, and Kruskal‐Wallis test with Dunn‐Bonferroni pairwise post‐hoc comparison for the thrombus location.

All variables that approached statistical significance in the univariate analysis (*P* < .1) were then evaluated iteratively as predictors in a multivariable binary logistic regression analysis for the TD versus NTD cases, and a multivariable multinomial regression analysis for comparison by thrombus location, using backward elimination approach. Breed and NIBP were excluded from the multinomial logistic regression because of the high degrees of freedom for breed (df = 16), and large number of missing cases for NIBP (n = 14). Results with a *P* value of ≤.05 were considered statistically significant.

## 3 RESULTS

### 3.1 TD cases

Four hundred seventy‐eight cases were flagged in the initial database search for dogs with TD, of which 84 had evidence of both TD and UPC > 2. However, only 50 dogs met the inclusion criteria; frequent reasons for exclusion included lack of a urine sediment exam (n = 11), presence of concurrent urinary tract infections (n = 5), and inconclusive imaging findings (n = 7). Of the 50 dogs included, 6 were male entire, 3 female entire, 19 male neutered, and 22 female neutered.

The most common thrombus site was the aorta (n = 20), followed by pulmonary vessels (n = 16), limb artery (n = 5), splenic vein (n = 3), vena cava (n = 3), portal vein (n = 3), renal vein (n = 2), jugular vein (n = 2), limb vein (n = 1), renal artery (n = 1), uterine arteries (n = 1).

37/50 cases had a recorded comorbidity; myxomatous mitral valve disease (MMVD; n = 6), adrenal mass (n = 6), acute kidney injury (AKI, n = 5; including 1 dog receiving dialysis), pancreatitis (n = 5), aspiration pneumonia (n = 4), urinary incontinence (n = 3), hyperadrenocorticism (n = 2), epilepsy (n = 2), allergic skin disease (n = 2), and 1 case each of IMHA, IMPA, IBD, pacemaker, craniomandibular osteopathy, Fanconi syndrome, lytic vertebral lesion, inflammatory tracheal mass, unspecified head mass, meningioma, hepatopathy, previous portosystemic shunt ligation, hypothyroidism, discospondylitis, arteritis, abscess, iatrogenic hyperadrenocorticism, diabetes mellitus, and unspecified splenic mass. Two cases were receiving orally administered glucocorticoid therapy.

Eight cases had renal histology performed, with findings including lymphoplasmacytic nephritis (n = 3), glomerulosclerosis (n = 2), membranoproliferative glomerulonephritis (n = 2), membranous glomerulopathy (n = 1), and no clinically relevant lesions (n = 2).

Five cases (10%) had a diagnosis of proteinuria before presenting with TD, 2 of which were diagnosed elsewhere and 3 of which had previously been evaluated at the QMHA; none of these cases were receiving antithrombotic medications.

### 3.2 NTD cases

The NTD group consisted of 100 dogs; 22 were male entire, 3 female entire, 39 male neutered, and 36 female neutered.

91/100 cases had a recorded comorbidity; hyperadrenocorticism (n = 13), MMVD (n = 10), unspecified cardiac murmur (n = 6), diabetes mellitus (n = 6), urinary incontinence (n = 5), inflammatory CNS disease (n = 5), IMPA (n = 3), lymphoma (n = 5), pancreatitis (n = 5), epilepsy (n = 4), adrenal mass (n = 4), unspecified hepatic mass (n = 4), allergic skin disease (n = 3), AKI (n = 2), anal sac adenocarcinoma (n = 2), IBD (n = 2), Fanconi syndrome (n = 2), pyothorax (n = 2), mast cell tumor (n = 2), and 1 each of hepatocellular carcinoma, septic arthritis, gall bladder mucocele, pacemaker, IMHA, previous pulmonary adenocarcinoma, squamous cell carcinoma, osteosarcoma, mammary adenoma, tonsillitis, hepatopathy, uveitis, patent ductus arteriosus, pulmonary fibrosis, chronic otitis, plasmacytoma, intervertebral disc extrusion, hypoadrenocorticism, hypothyroidism, chronic bronchitis, suspected hemangiosarcoma, GDV, gastrointestinal hemorrhage, nasal carcinoma, leishmaniasis, bronchopneumonia, cutaneous and renal glomerular vasculopathy (receiving plasmapheresis), renal dysplasia, previous portosystemic shunt, salivary mucocoele. 6/100 cases were receiving orally administered glucocorticoid therapy.

Seven cases had renal histology performed, with findings including glomeruloscerosis (3), interstitial nephritis (2), mild thickening of Bowmans capsule (1), severe amyloidosis (1), membranoproliferative glomerulonephritis (1), and glomerular vasculopathy (1).

Twelve of these NTD cases (12%) had a diagnosis of proteinuria before presenting to the QMHA, and 88 cases (88%) were diagnosed with proteinuria at the QMHA.

### 3.3 Comparison of TD vs NTD cases

Cavalier King Charles Spaniels (CKCS) and sighthounds were over‐represented in the TD group (*P* = .02; Table 1). No sex (50% of the dogs with TD were male and 61% of the NTD dogs) or neuter status (82% of the dogs with TD were neutered and 75% of the NTD dogs) predispositions were found. For individual comorbidities that were investigated (MMVD, AKI, hyperadrenocorticism, pancreatitis, adrenal masses, and all neoplasia) only the prevalence of AKI was different, being more common in dogs with TD (*P* = .04). There was no significant difference in the prevalence of orally administered glucocorticoid therapy between groups. The frequency of platelet clumping on blood smears was not significantly different between groups.

**TABLE 1:** Comparison of breed frequencies between dogs with renal proteinuria with (TD) and without (NTD) thrombotic disease.

		NTD	TD	Total
Breed	Bichon Frise	6 (6%)	1 (2%)	7
	**Cavalier King Charles Spaniel**	**1 (1%)**	**5 (10%)**	**6**
	Cocker Spaniel	11 (11%)	2 (4%)	13
	Crossbreed	14 (14%)	7 (14%)	21
	Jack Russell Terrier	5 (5%)	0 (0%)	5
	Labrador	5 (5%)	5 (10%)	10
	Large other breed	25 (25%)	9 (18%)	34
	Miniature Schnauzer	4 (4%)	2 (4%)	6
	**Sighthound**	**4 (4%)**	**7 (14%)**	**11**
	Small other breed	15 (15%)	9 (18%)	24
	West Highland White Terrier	4 (4%)	3 (6%)	7
	Yorkshire terrier	6 (16%)	0 (0%)	6
Total		100	50	150

*Note*: Bold text indicates a subset of categories whose column proportions differ significantly from each other at the .05 level.

Compared to NTD cases, TD cases had higher blood monocyte (*P* = .008) and neutrophil (*P* < .001) counts, and lower eosinophil counts (*P* < .001), serum potassium concentration (*P* = .02), total serum calcium concentration (*P* = .003), and serum albumin concentration (*P* = .003; Table 2).

**TABLE 2:** Comparison of continuous variables between dogs with renal proteinuria with (TD) and without (NTD) thrombotic disease.

Variable	NTD (n = 100)			TD (n = 50)			*P*
	n	Mean ± SD	Median [25th, 75th %]	n	Mean ± SD	Median [25th, 75th %]	
Age (years)	100		8.5 [6.3, 11.4]	50		10.1 [7.0, 12.0]	.15
**Albumin (g/dL)**	**89**	**2.83 ± 0.73**		**48**	**2.45 ± 0.62**		**.003**
Chloride (mmol/L)	89		111.0 [107.1, 114.0]	48		111.0 [105.6, 114.0]	.97
Cholesterol (mg/dL)	89		312 [232, 415]	46		323 [249, 426]	.53
Creatinine (mg/dL)	90		1.12 [0.75, 2.01]	48		1.19 [0.96, 1.95]	.29
Globulins (g/dL)	89	3.03 ± 0.66		48	2.85 ± 0.63		.13
**Potassium (mmol/L)**	**89**		**5.00 [4.61, 5.30]**	**48**		**4.80 [4.43, 5.10]**	**.02**
Sodium (mmol/L)	89		148.6 [146, 151]	48		149.0 [146.0, 151.4]	.65
**Total calcium (mmol/L)**	**89**		**2.47 [2.30, 2.70]**	**48**		**2.3 [2.02, 2.51]**	**.003**
Urea (mg/dL)	89		28.8 [14.3, 50.4]	48		25.6 [17.1, 56.9]	.50
Hematocrit (%)	86	42.6 ± 10.2		46	45.2 ± 10.7		.16
**Eosinophils (×10** ^**9**^ **/L)**	84		**0.17 [0.04, 0.41]**	46		**0 [0, 0.21]**	**<.001**
Lymphocytes (×10^9^/L)	84		1.23 [0.77, 1.90]	46		0.98 [0.61, 1.47]	.08
**Monocytes (×10** ^**9**^ **/L)**	**85**		**0.68 [0.42, 1.12]**	**46**		**1.02 [0.29, 1.57]**	**.008**
**Neutrophils (×10** ^**9**^ **/L)**	**84**		**7.31 [5.63, 11.06]**	**46**		**11.06 [8.92, 16.58]**	**<.001**
Urine protein: creatinine	100		4.11 [2.55, 6.63]	50		4.75 [2.93, 8.35]	.11
Noninvasive blood pressure (mm Hg)	86	170 ± 38		46	181 ± 40		.2

*Note*: The bold values indicates *p* < 0.05.

**FIGURE 1:**
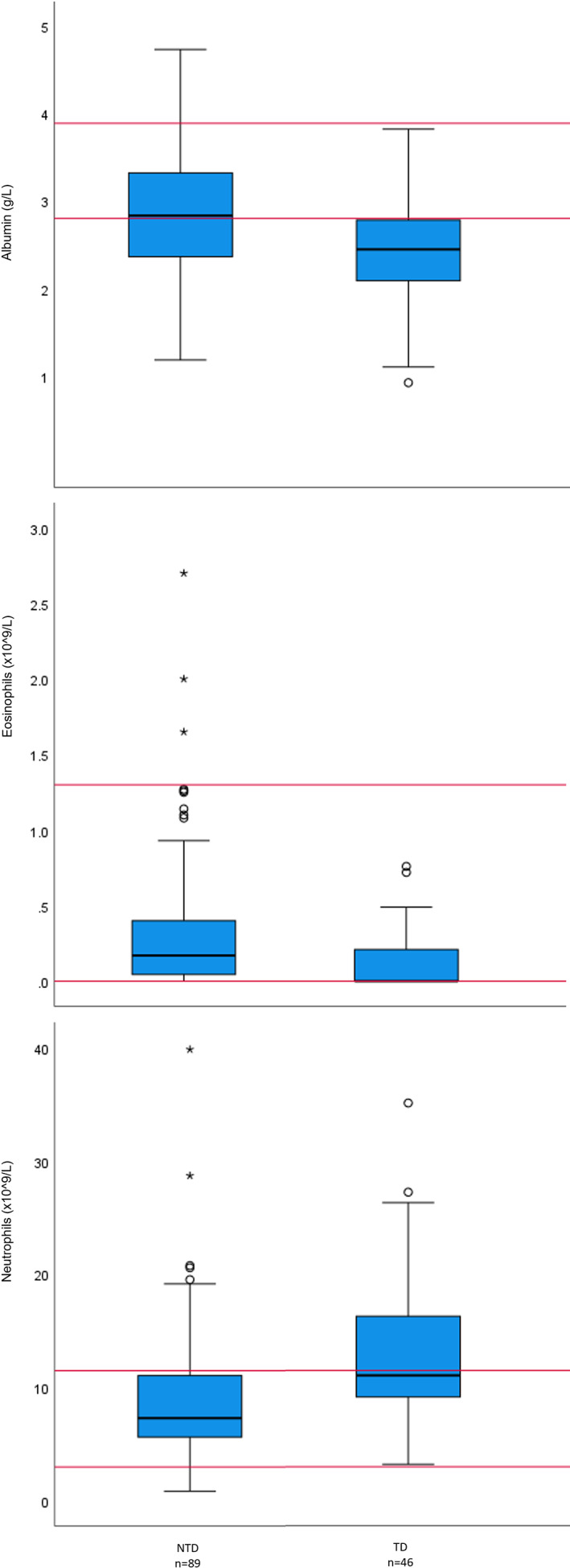
Box and whisker plots comparing variables which were significantly different between dogs with (TD) and without thrombotic disease (NTD) in a multivariable analysis. The red lines indicate the upper and lower extent of the reference interval for each variable.

Breed, presence of AKI, eosinophil count, hematocrit, neutrophil count, monocyte count, lymphocyte count, serum potassium concentration, total serum calcium concentration, and serum albumin concentration were included in the binary logistic regression evaluation. Albumin concentration (*P* = .04) and eosinophil count (*P* = .002) were independently and negatively associated with TD, neutrophil count was positively associated with TD (*P* = .02), and CKCS were independently at increased risk (OR = 98.8, 95% CI 2.09‐4671, *P* = .02; Table 3; Figure 1).

**TABLE 3:** Summary of variables that were significantly different between cases of dogs with renal proteinuria with (TD) and without (NTD) thrombotic disease in multivariable binary logistic regression analysis.

Variable	Odds ratio	95% CI	*P*
Albumin (g/dL)	0.93	0.87‐1.0	.04
Eosinophils (×10^9^/L)	0.06	0.009‐0.36	.002
Neutrophils (×10^9^/L)	1.09	1.01‐1.17	.02
Cavalier King Charles Spaniel breed	98.8	2.09‐4670	.02

Antithrombin activity was measured in 7 TD cases and 4 NTD cases. It was below the reference range in 1 TD case, and within the reference range for the remaining TD and all NTD cases.

### 3.4 Comparison by thrombus location

Ten cases had VT, 24 cases had AT, and 11 cases had PT. Five cases were excluded from this analysis because TD affected multiple locations; AT & PT (3), VT & PT (2).

In univariate analysis crossbreed dogs and Labradors were over‐represented for PT compared to AT, and Miniature Schnauzers were over‐represented for VT compared to AT (Table 4). No differences in sex or the prevalence of platelet clumping on blood smear, comorbidities or orally administered glucocorticoid use were detected between groups. A significant difference was found for neuter status between the 3 groups (*P* = .009). Dogs with VT were more likely to be entire (50%) than dogs with AT (13%) or PT (0%).

**TABLE 4:** Comparison of breed frequencies between dogs with renal proteinuria with venous, arterial or pulmonary thrombi.

		Thrombus site			Total
		Venous	Arterial	Pulmonary	
Breed	Bichon Frise	1 (10%)	0 (0%)	0 (0%)	1
	Cavalier King Charles Spaniel	0 (0%)	4 (16.7%)	0 (0%)	4
	Cocker Spaniel	1 (10%)	0 (0%)	1 (9.1%)	2
	**Crossbreed**	**1**_**a,b**_ **(10%)**	**1**_**b**_ **(4.2%)**	**4**_**a**_ **(36.4%)**	**6**
	**Labrador**	**1**_**a,b**_ **(10%)**	**1**_**b**_ **(4.2%)**	**3**_**a**_ **(27.3%)**	**5**
	Large other breed	2 (20%)	4 (16.7%)	1 (9.1%)	7
	**Miniature Schnauzer**	**2**_**a**_ **(20%)**	**0**_**b**_ **(0%)**	**0**_**a,b**_ **(0%)**	**2**
	Sighthound	0 (0%)	6 (25%)	1 (9.1%)	7
	Small other breed	2 (20%)	6 (25%)	1 (9.1%)	9
	West Highland White Terrier	0 (0%)	2 (8.3%)	0 (0%)	2
Total		10	24	11	45

*Note*: Bold text indicates a subset of categories whose column proportions differ significantly from each other at the .05 level. Each subscript letter denotes a subset of categories whose column proportions do not differ significantly, with columns not sharing a subscript letter having a significant difference.

VT cases had higher serum cholesterol concentration than dogs with PT (*P* = .04). AT had higher serum sodium concentration than dogs with VT (*P* = .03), higher age than dogs with PT (*P* = .02), and higher serum albumin concentration than both dogs in VT (*P* = .03) and PT (*P* = .008) groups (Table 5). There was an overall difference in hematocrit between groups (*P* = .04), but no differences were detected in the post‐hoc analysis.

**TABLE 5:** Comparison of continuous variables between dogs with renal proteinuria with venous, arterial or pulmonary thrombotic disease.

Variable	Venous (n = 10)			Arterial (n = 24)			Pulmonary (n = 11)			*P*
	n	Mean ± SD	Median [25th, 75th %]	n	Mean ± SD		n	Mean ± SD	Median [25th, 75th %]	
**Age (years)**	**10**	**9.1 ± 4.2**		**24**	**10.6 ± 2.6**		**11**	**7.0 ± 4.3**		**.02**
**Albumin (g/dL)**	**10**	**2.17 ± 0.49**		**23**	**2.73 ± 0.48**		**11**	**2.08 ± 0.76**		**.003**
Chloride (mmol/L)	10	109.3 ± 5.6		23	111.1 ± 7.1		11	109.3 ± 3.5		.63
**Cholesterol (mg/dL)**	**8**		**398 [309, 691]**	**23**		**313 [232, 402]**	**11**		**255 [154, 402]**	**.04**
Creatinine (mg/dL)	10		1.31 [0.95, 3.68]	23		1.2 [1.05, 1.97]	11		1.16 [0.85, 3.9]	.75
Globulins (g/dL)	10		3.17 [2.73, 3.75]	23		2.65 [2.25, 3.2]	11		2.26 [2.13, 3.25]	.08
Potassium (mmol/L)	10		4.97 [4.59, 5.19]	23		4.80 [4.40, 5.10]	11		4.70 [4.50, 5.10]	.73
**Sodium (mmol/L)**	**10**		**146.5 [142.3, 149.3]**	**23**		**151.0 [148.0, 153.0]**	**11**		**148.4 [145.8, 150.8]**	**.02**
Total calcium (mmol/L)	10	2.22 ± 0.63		23	2.37 ± 0.37		11	2.25 ± 0.35		.60
Urea (mg/dL)	10		40.6 [13.2, 89.3]	23		24.9 [18.5, 54.9]	11		51 [9, 58]	.92
Eosinophils (×10^9^/L)	9		0 [0, 0.14]	23		0 [0, 0.12]	10		0.11 [0, 0.43]	.51
**Hematocrit (%)**	**9**	**40.0 ± 10.2**		**23**	**49.3 ± 11**		**10**	**41.7 ± 8.7**		**.04**
Lymphocytes (×10^9^/L)	9		0.94 [0.65, 3.09]	23		0.86 [0.48, 1.22]	10		1.37 [0.77, 1.83]	.12
Monocytes (×10^9^/L)	9		1.36 [0.83, 1.80]	23		0.92 [0.43, 1.56]	11		1.02 [0.29, 1.46]	.46
Neutrophils (×10^9^/L)	9	14.84 ± 9.2		23	11.70 ± 5.90		11	13.07 ± 7.20		.52
Urine protein: creatinine	10		4.63 [3.88, 5.85]	24		4.62 [2.60, 9.50]	11		3.85 [2.81, 15.64]	.99
Noninvasive blood pressure (mm Hg)	5		160 [155, 208]	17		200 [170, 215]	9		160 [120, 198]	.85

*Note*: The bold values indicates *p* < 0.05.

**FIGURE 2:**
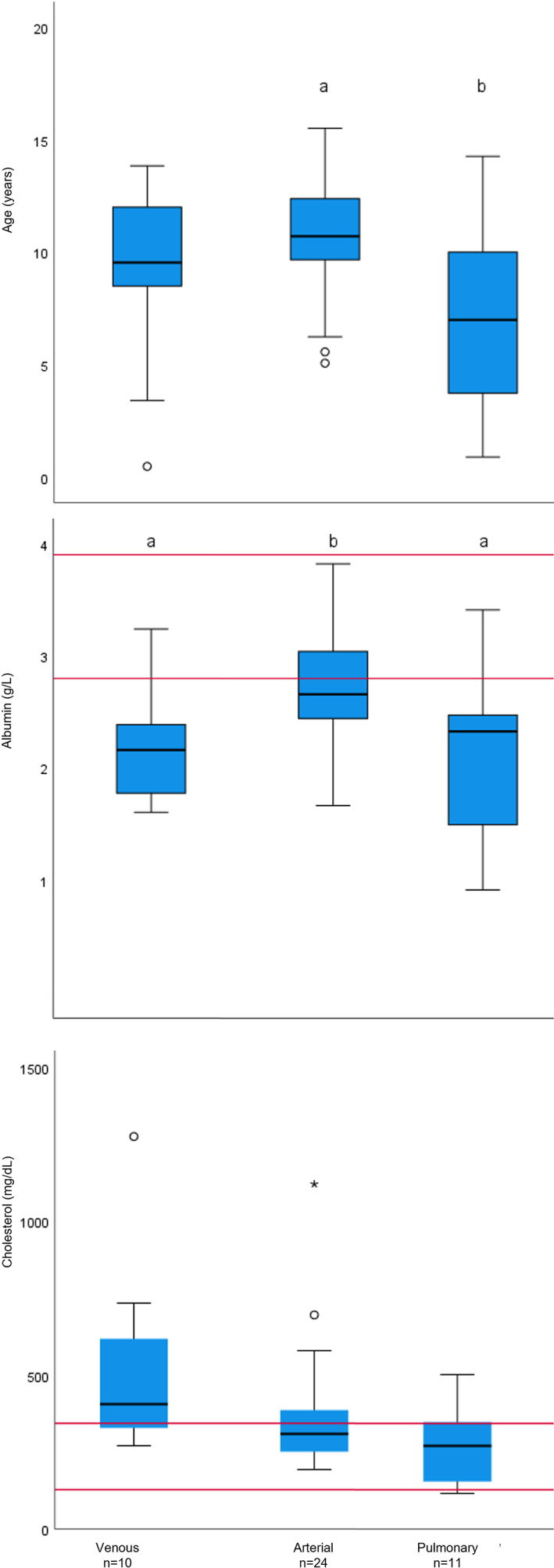
Box and whisker plots comparing variables which were significantly different between dogs with different locations of thrombotic disease in a multivariable analysis. The red lines indicate the upper and lower extent of the reference interval for each variable.

Neuter status, age, albumin, globulin, sodium, cholesterol, and hematocrit were included in the multivariable analysis. Overall, age, albumin, cholesterol, globulin, and neuter status were independent predictors of thrombus location. In the pairwise comparisons, AT had higher albumin than VT (*P* = .03) and higher age than PT (*P* = .008), and VT had higher age (*P* = .008) and lower cholesterol (*P* = .03) than PT (Figure 2).

## 4 DISCUSSION

Our study identified that CKCS with renal proteinuria had higher rates of TD compared to other breeds. CKCS have been identified as predisposed to AT, particularly aortic thrombosis, although this has not been specifically associated with PLN.^13^, ^14^ All 5 dogs of this breed with TD had AT (1 case had concurrent PT), 4 of which were aortic, although breed was not a significantly associated factor in the analysis of thrombus location. Reasons suggested for a genetic predisposition of CKCS for TD include a connective tissue disorder, a subset of the breed having increased platelet reactivity, and their increased incidence of cardiac disease.^13^, ^15^, ^16^ MMVD was present in 4 out of 5 of the CKCS with TD and was a common comorbidity in dogs of other breeds with TD in this study; however, the proportions of dogs with this comorbidity were not different between TD cases and NTD cases, and MMVD is not considered a risk factor for TD.^17^

Greyhounds are overrepresented in case series of aortic thrombosis.^10^ In this study, sighthounds (greyhounds, lurchers, and whippets) were associated with TD when breed was considered in isolation, but not in the multivariate analysis. Six of 7 sighthounds in the study had AT.

It is unclear whether of the association with TD in the context of proteinuria in these breeds is because of a difference in the underlying renal disease, or whether a breed related predisposition to TD in general is simply an additional risk factor. The lack of histopathological diagnoses in the majority of cases in this study prevents us from making this distinction. CKCS are not recognized as having a particular breed related cause of PLN, and whereas Greyhounds have a predisposition to cutaneous and renal glomerular vasculopathy this diagnosis was not made in any of our TD cases.^1^, ^18^

A variety of comorbidities were present in dogs both with and without TD in our study, with higher proportion of dogs without TD having a comorbidity. As this study reflects dogs seen in a referral hospital almost every dog will have had some underlying ailment to explain their presentation to the hospital. This result likely indicates that dogs in the NTD group were in many instances not presented for proteinuria but for a concurrent disease whereas the development of thrombi might have precipitated the referral of at least some of the dogs in the TD group. In the present study both hyperadrenocorticism and diabetes were more common in the NTD group than in dogs with TD. This is unexpected for hyperadrenocorticism as this disease is considered associated with TD in dogs, especially in the presence of other comorbidities^4^; this might have been because of selection bias, with cases of hyperadrenocorticism and TD not being flagged on our keyword search if clinicians did not comment on (or test for) proteinuria. Diabetes is associated with proteinuria in dogs but is not reported as a cause of TD, whereas in humans diabetes is associated with TD in patients both with or without PLN.^19^, ^20^, ^21^, ^22^, ^23^ AKI was the only comorbidity over‐represented in dogs with TD in the univariate analysis, and this did not remain significant in the multivariate analysis. In humans AKI has been associated with an increased risk of VT in people with nephrotic syndrome, and appears to be because of inflammation causing further hypercoagulability.^24^, ^25^

In our study, TD was associated with lower serum albumin concentrations. The latter finding is demonstrated in dogs in some but not all studies.^2^ The large overlap in albumin concentrations between dogs with and without TD means care should be taken when extrapolating from this observation. However, dogs with normal serum albumin concentrations might be considered at relatively low risk of thrombotic complications, with 73% of TD cases having a serum albumin concentration below the laboratory reference interval compared to 38% of cases without TD. Our finding of significantly lower serum albumin concentrations in dogs with VT compared to AT suggests hypoalbuminemia is more important in the pathogenesis of the former. Hypoalbuminemia is identified as a risk factor for VT in human patients with nephrotic syndrome,^26^, ^27^ in some but not all studies.^28^, ^29^, ^30^ Low serum albumin is not associated with AT.^27^ The association between TD and hypoalbuminemia in PLN is frequently suggested to be because of the correlation between renal losses of albumin and antithrombin, with deficiency of the latter leading to hypercoagulability.^31^ In our study antithrombin activity was only below the reference range in 1 out of the 7 TD cases where this was measured, suggesting this is unlikely to be the primary cause of hypercoagulability. There is no significant association between low antithrombin activity and TD in dogs with PLN.^6^ Another indirect explanation for the association is that as a negative acute phase protein albumin concentrations are lowered in inflammatory states, and inflammation (which will be present in many causes of PLN) predisposes to TD.^32^, ^33^

Hypoalbuminemia was also a risk factor for TD in a study of dogs with immune mediated hemolytic anemia, so decreased serum albumin concentration might directly increase TD risk rather than simply correlating to other risk factors or being a marker of more severe disease.^34^ A suggested mechanism for this is that low serum albumin concentrations increase platelet sensitivity to activation, as in dogs and humans.^35^, ^36^

Increased UPC was not associated with TD in our study which suggests this should not be used to predict TD risk, however a previous study did find this association in dogs.^2^ Human studies do not show an association between degree of urine protein loss and TD.^26^

Dogs with TD in our study had significantly higher total leukocyte counts, neutrophils and monocytes than those without, and lower eosinophils. This could simply reflect presence of a stress leukogram as a result of TD. However, these changes could also be the result of inflammatory processes, which have been documented to result in a hypercoagulable state in dogs.^37^ Leukocytosis has been identified as a risk factor for TD in humans with cancer and chronic myeloproliferative diseases.^38^, ^39^ Leukocytosis has been correlated with the presence of ischemic lesions at necropsy in dogs with IMHA, and this was thought likely to be because of TD.^40^

In our study, the most common location for thrombi was the aorta, followed by the PT. The CURATIVE consensus statement concluded the PT appeared to be the most common location for thrombi in dogs with PLN, followed by aortic thrombi.^4^ In humans with nephrotic syndrome, VT is considered to be the most common site for TD in adults, with AT more frequently seen in children.^31^ It is possible that PT were under‐represented in our study because of the difficulty in diagnosing these ante‐mortem.

Entire dogs were overrepresented for VT in this study. Given the higher incidence of AT in children this raises the possibility that sex hormones might influence TD predilection sites. In humans, hormonal contraceptives and hormone replacement therapy,^41^ and testosterone therapy in predisposed individuals,^42^ increases the risk of VT. However, data on neuter status in dogs should be interpreted with caution because listing of dogs as entire is often an error of omission on medical records which often require a box to be ticked to indicate that a dog has been neutered.

We found that dogs with AT and VT were older than those with PT, although this did not remain significant in the multivariate analysis. This reflects the situation in humans, where age was a predictor of AT over VT and PT for adults with nephrotic syndrome.^27^ Age, hypertension, and other risk factors identified in that study such as smoking and diabetes, are also risk factors for atherosclerosis leading the authors to conclude there might be a different pathophysiological mechanism for the types of TD in their study cohort. While atherosclerosis itself is rare in dogs, it is possible that reduced arterial elasticity and hypertension in older dogs could lead to local endothelial dysfunction that could act as a nidus for thrombus formation.^43^, ^44^, ^45^, ^46^, ^47^

VT was associated with increased serum cholesterol concentrations compared to PT. Hypercholesterolemia is recognized to occur in many dogs with PLN and as part of the “nephrotic syndrome” that some will develop.^1^ In humans, serum lipoprotein(a) levels correlate with total cholesterol levels and are known to increase risk of TD.^48^, ^49^ However, much of this increased risk is because of impact lipoprotein(a) has on the development of atherosclerosis; this is a rare condition in dogs, and would be expected to affect the arterial system.^43^ Lipoprotein(a) also directly promotes TD by inhibiting the activation of plasminogen, reducing fibrinolytic activity, and decreased fibrinolysis has been associated with VT in humans.^49^, ^50^

An important limitation of this study is the lack of a histological diagnosis for the renal disease in the majority of cases, with renal histology performed in only 8 TD cases and 7 NTD cases. In humans it is suggested the risk of TD varies between different histological diagnoses.^51^ As renal biopsies are not frequently performed in practice it is still useful to attempt to classify dogs with routine blood tests and signalment, but further work to investigate the underlying mechanisms behind the differences we have highlighted are warranted to help develop more effective treatment recommendations.

The retrospective nature of the study was another limitation as there was no standardization of treatment or investigation, which could have influenced some of the findings. Persistence of the proteinuria was not confirmed in many cases; this was not made an inclusion criteria as case numbers would have been limited by exclusion of cases with thrombi that did not survive their initial visit (30% of TD cases), but this meant that some of our cases might have had transient proteinuria because of systemic disease rather than primary glomerular disease.

A major limitation of the study was the potential for misclassification of some NTD cases. Post‐mortem examination was only performed on 4 NTD dogs, compared to 7 TD cases, so it is possible that some cases had undocumented TD. However, all thrombi that were documented post‐mortem had either been previously identified on diagnostic imaging or had been clinically suspected (3 cases of pulmonary thrombi), so we consider the frequency of undetected thrombi to be relatively low. Similarly, a higher proportion of TD cases had CT scans performed (34%) compared to NTD cases (26%), and it is possible that thrombi might have been detected in the NTD group if they had been screened with this more sensitive imaging modality. Imaging modalities were chosen based on clinical indication, and varied over time as availability of advanced imaging improved, but we cannot exclude the possibility that some NTD cases were misclassified.

Small numbers of different individual breeds and comorbidities mean our study was likely underpowered to detect small differences.

In conclusion, our study identifies several factors associated with the development of TD in dogs with renal proteinuria. In addition, we identified different factors associated with the development of VT, AT, and PT, suggesting there might be differences in the underlying disease processes.

## CONFLICT OF INTEREST DECLARATION

Neither author received funding that is directly related to the material presented in this paper. One author has received financial support for a research group (unrelated to this work), as well as consultancies and speakers honoraria from Royal Canin, Mars Petcare, Hill's, Idexx, Boehringer Ingelheim, Vetoquinol, MSD, Pfizer, Elanco, and PetPlan Charitable Trust.

## OFF‐LABEL ANTIMICROBIAL DECLARATION

Authors declare no off‐label use of antimicrobials.

## INSTITUTIONAL ANIMAL CARE AND USE COMMITTEE (IACUC) OR OTHER APPROVAL DECLARATION

Authors declare no IACUC or other approval was needed.

## HUMAN ETHICS APPROVAL DECLARATION

Authors declare human ethics approval was not needed for this study.
